# Response patterns and impact of MRD in patients with *IDH1/2*-mutated AML treated with venetoclax and hypomethylating agents

**DOI:** 10.1038/s41408-023-00915-6

**Published:** 2023-09-21

**Authors:** Danielle Hammond, Sanam Loghavi, Sa A. Wang, Marina Y. Konopleva, Tapan M. Kadia, Naval G. Daver, Maro Ohanian, Ghayas C. Issa, Yesid Alvarado, Nicholas J. Short, Koji Sasaki, Naveen Pemmaraju, Guillermo Montalban-Bravo, Curtis A. Lachowiez, Abhishek Maiti, Guillermo Garcia-Manero, Elias J. Jabbour, Gautam Borthakur, Farhad Ravandi, Koichi Takahashi, Sherry R. Pierce, Hagop M. Kantarjian, Courtney D. DiNardo

**Affiliations:** 1 Department of Leukemia, The University of Texas M.D. Anderson Cancer Center, Houston, TX USA; 2 Department of Hematopathology, The University of Texas M.D. Anderson Cancer Center, Houston, TX USA

**Keywords:** Acute myeloid leukaemia, Drug development

Several lower-intensity therapies for isocitrate dehydrogenase 1/2 (*IDH1/2*)-mutated AML are available (Supplemental Table 1), including small molecule inhibitors of mutant IDH1 (ivosidenib and olutasidenib) and IDH2 (enasidenib), used alone or in combination with broader anti-leukemic agents. While not molecularly targeted therapy per se, venetoclax-based therapy has preferential activity in *IDH1/2*-mutated disease [1–3]. Given uncertainty surrounding the optimal sequence of these agents, we investigated the quality and duration of responses to venetoclax and hypomethylating agent (VEN + HMA) combinations in *IDH1/2*-mutated AML. We additionally characterize response patterns in a subset of these patients who were consecutively treated (either directly before or after VEN + HMA) with IDH inhibitor (IDHi)-based regimens.

Patients with newly diagnosed (ND) or relapsed/refractory (R/R) AML with *IDH1*/2 mutations (*IDH*^mut^) who received ≥1 cycle of azacitidine or decitabine with VEN at our institution were included, irrespective of prior exposure to these agents. Within-patient responses were then evaluated in a subset of these patients who consecutively received IDHi-based regimens as an alternate line of therapy (LoT). Change in treatment for lack of optimal response (including persistent or emergent measurable residual disease [MRD]) was considered a separate LoT.

Responses were evaluated per 2017 ELN criteria [4]. MRD evaluation by 8-color multiparameter flow cytometry (FC-MRD, limit of detection [LoD] 0.1–0.01%) was also performed and used to determine “best” response in patients who attained complete remission or complete remission with incomplete count recovery (CR/CRi), i.e., the time of best response was upon attaining FC-MRD negativity in applicable patients. *IDH*^mut^ variant allele frequencies (VAFs) from bone marrow samples were assessed pre-treatment and at the time of best response and analyzed by either Sanger sequencing (SS, LoD 10–20%) or amplicon-based next-generation sequencing (NGS, LoD 2%). As the sensitivity of SS is insufficient for genomic MRD assessment, digital droplet polymerase chain reaction (ddPCR, LoD 0.1–0.5%) was retrospectively performed on samples with no available *IDH*^mut^ sequencing or a negative SS result at the best response. An undetectable *IDH*^mut^ result from either NGS or ddPCR was considered genomic MRD (NGS-MRD) negative, whereas only a negative result by ddPCR was defined as molecular clearance.

Overall survival (OS) for the primary analysis was calculated from C1D1 of VEN + HMA until death or the last follow-up date. Duration of response (DoR) was calculated from the date of best response until relapse, receipt of subsequent antileukemia therapy, or death without censoring for allogeneic stem cell transplant (alloSCT). Relationships between response and categorical pre-treatment characteristics were evaluated using a two-tailed Fisher exact test. Comparative time-to-event analyses were assessed using the log-rank method.

Sixty-five patients were included (Supplemental Table 2). Forty-five percent of patients had ELN adverse risk disease, and 22% had a monocytic (French-American-British M4 or M5) phenotype. Therapy-related AML (5%) and complex cytogenetics (18%) were infrequent. Seventy percent of cases (*n* = 46) were *IDH2*-mutated (Supplemental Fig. 1); one patient had concurrent *IDH1 R132C* and *IDH2 R140Q* mutations, the latter emerging on antecedent treatment with ivosidenib. The co-mutation landscape was enriched for *SRSF2* (42%), *NPM1* (28%), and epigenetically relevant mutations (Supplemental Fig. 2).

The study population was heterogeneously treated (Supplemental Table 3). Fifty-five percent of patients received VEN + HMA in the frontline setting. A median number of VEN + HMA cycles was 3, with 50% and 45% of patients discontinuing therapy due to inadequate (11% ND, 24% R/R) or loss of (39% ND, 21% R/R) response. One-quarter of patients who discontinued VEN + HMA did so to proceed to alloSCT. Half (*n* = 33, 51%) of the VEN + HMA treated patients received IDHi-based regimens as alternate lines of AML therapy. Three of the 33 patients received multiple IDHi-based LoT at alternate time points, leading to 36 IDHi-based treatment courses. Only the 28 IDHi-based treatment courses received immediately preceding or following VEN + HMA therapy were analyzed (Supplemental Fig. 3).

Responses with VEN + HMA are shown in Fig. 1A. In the 36 ND patients, the CR/CRi rate was 86%, of which 93% and 32% of responses were FC-MRD and NGS-MRD negative, with a median DoR of 24.1 months (95% CI: 17.3–not estimable [NE]). In the 29 R/R patients, CR/CRi rate was 45% with a DoR of 15 months (95% CI: 6.0–NE), with comparable rates of FC-MRD (82%) and NGS-MRD (36%) negativity. While 90% of all CR/CRi responses were FC-MRD negative, only 33% were NGS-MRD negative. Out of the 44 CR/CRi responses, FC-MRD and NGS-MRD were not evaluable in 3 and 5 patients, respectively. Genomic MRD assessment of the *IDH*^mut^ was performed by NGS prospectively (*n* = 27) and ddPCR retrospectively (*n* = 12).

**Fig. 1:**
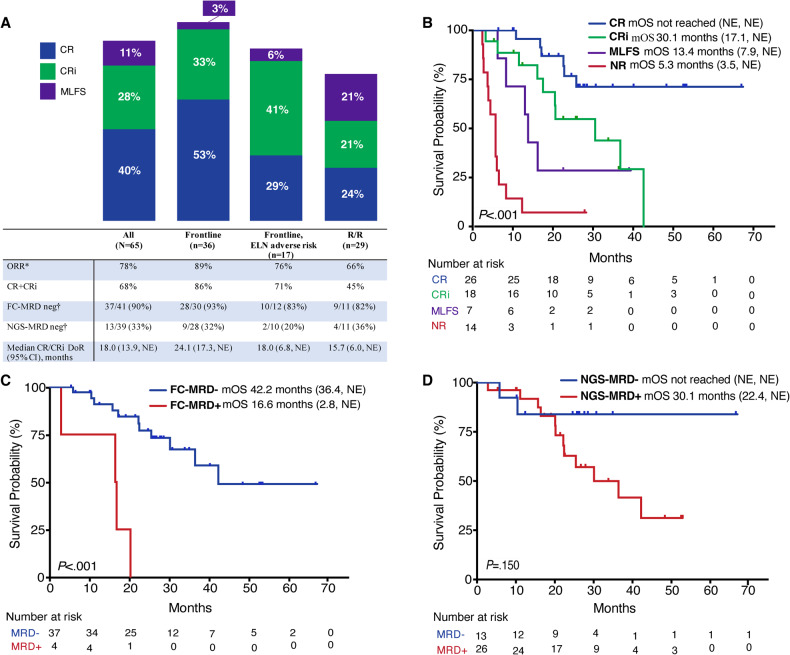
Outcomes with VEN + HMA. **A** Response outcomes. **B** Overall survival by type of marrow response (*N* = 65). Overall survival by FC-MRD (**C**) and NGS-MRD (**D**) status in patients with CR/CRi responses and evaluable MRD (*n* = 41 and *n* = 39, respectively). DoR duration of response, NE not estimable, ORR overall response rate. *ORR = CR + CRi + MLFS. †Of CR/CRi responses with evaluable MRD by the respective technique.

After a median follow-up of 17.3 months, median OS (mOS) was 42.2 months (95% CI: 25.4–NE) and 15.8 months (95% CI: 5.8–NE) in the ND and R/R cohorts, respectively (Supplemental Fig. 4). Repeating the survival analysis with censoring at time of alloSCT demonstrated a mOS of 42.2 months (95% CI: 20.2–NE) and 6.1 months (95% CI: 2.4–NE) in the ND and R/R cohorts, without a statistically significant difference from the original survival estimates (*P* = 0.850 ND, *P* = 0.386 R/R), although a trend toward decreased OS upon censoring for alloSCT in the R/R setting was observed. Quality of response was associated with a survival benefit (*P* < 0.001, Fig. 1B). While FC-MRD negativity in CR/CRi was significantly associated with an OS benefit (*P* < 0.001, Fig. 1C), NGS-MRD negativity was not despite a trend towards improved OS (*P* = 0.150, Fig. 1D).

Supplemental Fig. 5 summarizes the relationship between co-mutation landscape and response. *NPM1* co-mutations were associated with an increased likelihood of CR/CRi (89% *NPM1* mutated vs. 61% *NPM1* wildtype, OR 5.14, 95% CI: 1.05–25.09, *P* = 0.037) and improved OS (mOS not reached vs. 20.1 months, *P* = 0.048). Presence of ≥1 receptor tyrosine kinase (RTK) signaling co-mutations was associated with a lower likelihood of CR/CRi (50% RTK group vs. 79% non-RTK group, OR 0.27, 95% CI: 0.08–0.83, *P* = 0.025) and worse OS (mOS 11.1 months vs. 36.4 months, *P* = 0.006). Presence of a *TP53* mutation and/or deletion 17p was infrequent overall, but associated with a trend toward decreased likelihood of response (CR/CRi 45% *TP53*-mutated/deletion 17p vs. 75% *TP53* intact, *P* = 0.077) and a significantly worse OS (11.9 months vs. 30.1 months, *P* = 0.031). In ND patients, the likelihood of attaining CR/CRi and mOS appeared to be higher in *IDH2*- versus *IDH1*-mutated disease (CR/CRi 95% vs. 75%, OR 6.33, 95% CI: 0.63–63.64, *P* = 0.085; mOS not reached [95% CI: 30.1–NE] vs. 20.2 [95% CI: 11.1–NE] months, *P* = 0.063). There was no significant relationship between the likelihood of response nor OS with pre-treatment *IDH*^mut^ VAF and monocytic phenotype, respectively.

Figure 2 summarizes the sequential outcomes of VEN + HMA and IDHi-based regimens. A salvage response was defined as any morphologic response (including partial response and morphologic leukemia-free state) following lack/loss of response to the preceding regimen. In patients who switched from VEN + HMA to IDHi-based regimens (VEN + HMA > IDHi) due to lack/loss of response, the response rate was 56% (10/18 cases), and 2 patients were bridged to alloSCT. A higher salvage response rate (86%, 6/7 cases) was observed when an IDHi was added to VEN + HMA to create a triplet rather than switching to an IDHi + HMA doublet (29%, 2/7 cases) or IDHi monotherapy (50%, 2/4 cases). It is important to note that in 3 of the 7 cases, an IDHi was added in response to rising MRD rather than overt morphologic relapse. Two additional responding patients who transitioned from VEN + HMA to IDHi-based therapy for reasons other than loss/lack of response were annotated as maintenance of an existing response (Supplemental Table 4) and excluded from most salvage therapy sequence analyses. Conversely, 88% (7/8) of the cases in which an IDHi-based regimen was switched to VEN + HMA (IDHi > VEN + HMA) had a salvage response, and 4 patients were bridged to alloSCT (Supplemental Table 5). Strikingly, one patient who switched from enasidenib to VEN + HMA as seventh-line therapy attained an FC- and NGS-MRD negative CR. The mOS from C1D1 of whichever therapy was received first was 47.1 months (95% CI: 13.9–NE) in the IDHi > VEN + HMA group and 20.1 months (95% CI: 15.8–NE) in the VEN + HMA > IDHi group. There was an observed trend towards a longer mOS in patients who switched from VEN + HMA to a VEN + HMA + IDHi triplet rather than a subsequent IDHi doublet or monotherapy (Supplemental Fig. 6).

**Fig. 2:**
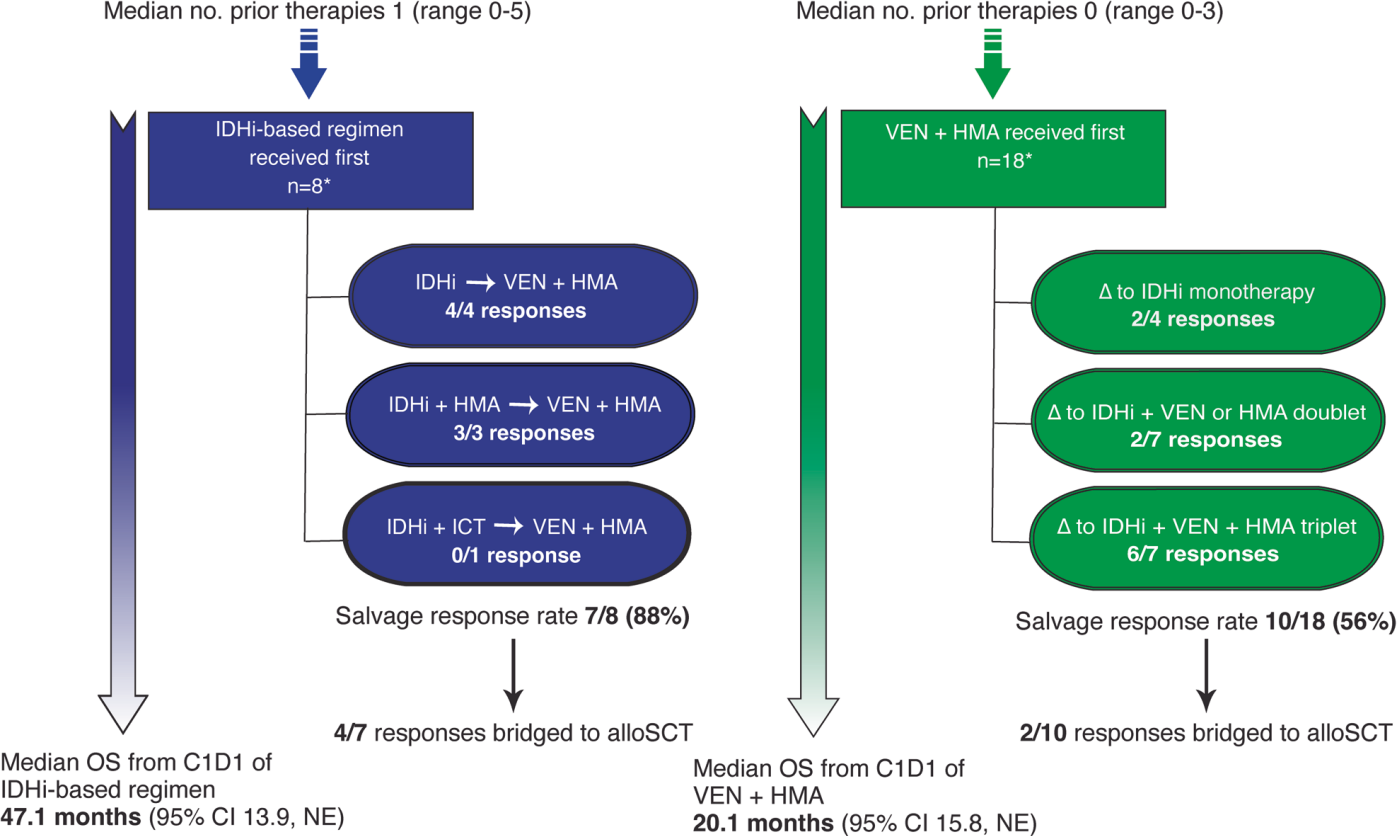
Within-patient response rates and survival by therapy sequence when switching between VEN + HMA and IDHi-based regimens due to lack/secondary loss of response. A salvage response was defined as any of CR, CRi, PR, or MLFS. *One patient was included in both groups for separate IDHi-based lines of therapy that immediately preceded and followed, respectively, their VEN + HMA treatment course.

In conclusion, we confirm that the combination of VEN + HMA results in high rates of durable remission and impressive overall survival in both ND and R/R *IDH1/2*-mutated AML, establishing a benchmark of outcomes with standard non-targeted therapy for AML in patients ineligible for high-dose chemotherapy [5]. While almost 90% of responses were MRD negative by flow cytometry, the *IDH*^mut^ remained detectable in most cases. MRD negativity by flow cytometry was associated with a statistically significant survival benefit. While hypothesis-generating at best, our secondary analysis of the sequential receipt of IDH inhibitors in a subset of these VEN + HMA treated patients is concordant with a recent prospective study reporting high rates of durable responses with VEN + HMA + IDHi combination therapy [6].

### Supplementary information

Supplemental Information

## Data Availability

Original data will not be publicly available. For special inquiries, please contact cdinardo@mdanderson.org.
