# Supplementary material for: Response patterns and impact of MRD in patients with IDH1/2-mutated AML treated with venetoclax and hypomethylating agents
Source: Blood Cancer J. 2023 Sep 21;13(1):148. doi: 10.1038/s41408-023-00915-6 (PMC10514257; doi:10.1038/s41408-023-00915-6)
Supplement: Supplementary file 1 — Supplemental Information [file 41408_2023_915_MOESM1_ESM.pdf]

Supplemental Information

Supplement to: Hammond D, Loghavi S, Konopleva MY, Kadia TM, Daver N, Ohanian M, et al. Response patterns and impact of MRD in patients with *IDH1/2*-mutated AML treated with venetoclax and hypomethylating agents

Contents

**Supplemental Tables**	**page 2**
**Supplemental Figures**	**page 7**

Supplemental Tables

Supplemental Table 1. Reported response rates and mutation clearance in newly diagnosed *IDH1/2*-mutated AML in patients ineligible for intensive chemotherapy

	Azacitidine	Venetoclax	Enasidenib	Ivosidenib	cCR (%)	*IDH* MC (%)*	Ref
Single agent	**A**				10.7	NR	Pollyea et al.[i]
			**Ena**		21.0	NR	Pollyea et al.[ii]
				**Ivo**	42.4	64	Roboz et al.[iii]
Doublet	**A**	**V**			79.0	NR	Pollyea et al.[i]
	**A**		**Ena**		53.0	NR	DiNardo et al.[iv]
	**A**			**Ivo**	69.6, 54.0†	69, 52	DiNardo et al.[v], Montesinos et al.[vi]
		**V**		**Ivo**	100.0	25	Lachowiez et al.[vii]
Triplet	**A**	**V**		**Ivo**	89.0‡	57‡	Lachowiez et al.[vii]

A azacitidine, *cCR* composite CR (CR + CRi + CRh), *Ena* enasidenib, *Ivo* ivosidenib, *NR* not reported, *MC* molecular clearance, *V* venetoclax.
*Reported as a percentage of responding patients with evaluable samples, performed by ddPCR with a VAF limit of detection 0.02-0.04% or <1% depending on the study to qualify as molecular clearance.
†CR + CRi rate reported in this case.
‡Outcomes shown in the cohort treated with the venetoclax 400mg dose level but a venetoclax 800mg dose level is being explored in the ongoing phase 1b/2 study.

i. Pollyea DA, DiNardo CD, Arellano ML, Pigneux A, Fiedler W, Konopleva M, et al. Impact of Venetoclax and Azacitidine in Treatment-Naive Patients with Acute Myeloid Leukemia and IDH1/2 Mutations. Clin Cancer Res. 2022;28(13):2753-61.
ii. Pollyea DA, Tallman MS, de Botton S, Kantarjian HM, Collins R, Stein AS, et al. Enasidenib, an inhibitor of mutant IDH2 proteins, induces durable remissions in older patients with newly diagnosed acute myeloid leukemia. Leukemia. 2019;33(11):2575-84.
iii. Roboz GJ, DiNardo CD, Stein EM, de Botton S, Mims AS, Prince GT, et al. Ivosidenib induces deep durable remissions in patients with newly diagnosed IDH1-mutant acute myeloid leukemia. Blood. 2020;135(7):463-71.
iv. DiNardo CD, Schuh AC, Stein EM, Montesinos P, Wei AH, de Botton S, et al. Enasidenib plus azacitidine versus azacitidine alone in patients with newly diagnosed, mutant-IDH2 acute myeloid leukaemia (AG221-AML-005): a single-arm, phase 1b and randomised, phase 2 trial. Lancet Oncol. 2021.
v. DiNardo CD, Stein AS, Stein EM, Fathi AT, Frankfurt O, Schuh AC, et al. Mutant isocitrate dehydrogenase 1 inhibitor ivosidenib in combination with azacitidine for newly diagnosed acute myeloid leukemia. J Clin Oncol. 2021;39(1):57-65.
vi. Montesinos P, Recher C, Vives S, Zarzycka E, Wang J, Bertani G, et al. Ivosidenib and Azacitidine in IDH1-Mutated Acute Myeloid Leukemia. N Engl J Med. 2022;386(16):1519-31.
vii. Lachowiez CA, Loghavi S, Zeng Z, Tanaka T, Kim YJ, Uryu H, et al. A phase Ib/II study of ivosidenib with venetoclax +/- azacitidine in IDH1-mutated myeloid malignancies. Blood Cancer Discov. 2023.

Supplemental Table 2. Patient and disease characteristics at time of starting VEN + HMA

	**N=65 patients**
**All patients**	
Median age (range), y	69 (34-87)
Age ≥75 y, n (%)	17 (26)
**Treatment-naïve patients (n=36)**	
Median age (range), y	72 (41-83)
Age ≥75 y, n (%)	12 (33)
**Male, n (%)**	41 (63)
**ECOG Performance status, n (%)**	
0-1	46 (71)
2	16 (25)
≥3	3 (5)
**WHO 2016 subtype at diagnosis, n, (%)**	
AML-MRC	24 (37)
t-AML	3 (5)
AML with mutated NPM1	12 (18)
AML with mutated RUNX1	4 (6)
AML NOS	22 (34)
**FAB M4/M5 subtype at diagnosis, n (%)***	14 (22)
**ELN 2017 risk group, n (%)**	
Favorable	14 (22)
Intermediate	16 (25)
Adverse	29 (45)
Not evaluable	6 (9)
**Cytogenetics, n (%)**	
Diploid	24 (37)
+8	8 (12)
Del(17p)	2 (3)
-7/-7q and/or -5/5q	9 (14)
Other	20 (31)
Complex cytogenetics	12 (18)
ELN adverse CG risk	15 (23)
Not evaluable	2 (3)
***IDH* mutation, n (%)**	
IDH1	19 (29)
IDH2	45 (69)
Both‡	1 (2)
**Pre-treatment *IDH*^{mut} VAF %, median (IQR)**	30 (14-44)
**Prior allogeneic SCT, n (%)**	7 (11)
**Bone marrow blasts, median (range)†**	42 (3-95)
**Peripheral blood counts, median (range)**	
White blood cells, 10^9/L	2.4 (0.1-81.1)
Absolute neutrophil count, 10^9/L	0.5 (0.0-10.3)
Hemoglobin, g/dL	9.5 (7.0-13.1)
Platelets, 10^9/L	60 (4-409)

CG cytogenetic, *ECOG* Eastern Cooperative Oncology Group, *ELN* European Leukemia Network, *FAB* French-American-British, *IQR* interquartile range, *SCT* stem cell transplant, *WHO* World Health Organization.
*FAB subtype was not evaluable in 1 patient.
†This range includes marrow blasts <20% as some patients were started on VEN + HMA for relapsed disease.
‡One patient had an original *IDH1* R132C mutation but acquired a dominant *IDH2* R140Q mutation on treatment with ivosidenib; both mutations were therefore present upon starting VEN + HMA. The patient was annotated as *IDH2*-mutated for subsequent analyses.

Supplemental Table 3. Treatment characteristics – VEN + HMA

	**N=65 patients**
**Median no. of prior AML therapies (range)***	0 (0-6)
**No. of prior AML therapies, n (%)**	
0	36 (55)
1	10 (15)
2	9 (14)
≥3	10 (15)
**Received prior ICT for AML, n (%)†**	25 (86)
**HMA combined with VEN, n (%)**	
Decitabine	55 (85)
Azacitidine	10 (15)
**Concurrent use of a FLT3 inhibitor, n (%)**	5 (8)
Sorafenib	3
Gilteritinib	2
**Administered on protocol, n (%)**	44 (68)
**Median no. of cycles (IQR)**	3 (2-10)
**Primary reason for discontinuation, n (%)‡**	
Inadequate response	10 (18)
Loss of response	17 (30)
Infection or other toxicity	3 (6)
Allogeneic SCT	14 (25)
Patient/MD choice	11 (20)
Died in CR/CRi	2 (4)
**Relative sequencing of an IDHi, n (%)§**	
Never received an IDHi at any timepoint	32 (47)
Received an IDHi prior to VEN + HMA	10 (15)
Received an IDHi after VEN + HMA	26 (38)

ICT intensive chemotherapy, *SCT* stem cell transplant.
*Therapies received for antecedent chronic-phase myeloid malignancies were not counted in the number of previous lines of therapy.
†Percentage of the n=29 patients who received prior therapy.
‡Percentage of the n=57 patients who discontinued VEN + HMA.
§Three patients each received two separate IDHi-containing regimens at different timepoints, accounting for 36 IDHi-based treatment courses in 33 patients.

Supplemental Table 4. Outcomes with IDH inhibitor-based regimens received directly post VEN + HMA (n=20)

	IDH^{mut}	No. prior LoT	Response VEN+HMA, Marrow/FC-/NGS-MRD[a]	ToT VEN + HMA, months	Reason for switch to IDHi	IDHi regimen	Response IDHi, Marrow/FC-/NGS-MRD[a]	Did the regimen salvage response post VEN+HMA[b]	ToT IDHi regimen, months[c]	Reason for IDHi regimen discontinuation
Δ to IDHi	1	0	CRi/Neg/Pos	14.8	LoR	Novel IDHi[d]	NR	No	0.7	NR
	2	1	CRi/NE/Pos	3.3	LoR	Ena	PR	Yes	8.2	LOR
	1	0	NR	4.0	NR	Novel IDHi[d]	NR	No	2.5	NR
	2	0	CR/Neg/Pos	2.7	Adverse event[e]	Ena	CR/Neg/Pos	Maintained	7.3+	
	2	1	MLFS	10.3	LoR	Ena[f]	CR/Neg/Neg	Yes	28.1+	
Δ to IDHi doublet	2	3	NR	3.5	NR	Ena+D	NR	No	1.1	NR
	2	1	NR	0.9	NR	Ena+D	CR/Neg/Neg	Yes	3.5	Patient choice[g]
	2	2	MLFS	2.7	LoR	Ena+V	NR	No	3.0	NR
	2	0	CRi/Neg/Pos	19.0	LoR	Ena+A	NR	No	2.1	NR
	2	0	MLFS	7.3	LoR	Ena+A	SD	No	5.0	NR
	2	2	NR	3.4	NR	Ena+A	NR	No	1.0	NR
	2	1	MLFS	2.4	LoR	Ena+A	MLFS	Yes	6.5	SCT
Δ to IDHi+ VEN+ HMA	2	1	CR/Pos/NE	10.7	LoR	Ena+V+A	CR/Pos/Pos	Yes	4.7	LOR
	2	0	CR/Neg/NE	29.6	LoR	Ena+V+A	CR/Neg/Pos	Yes	10.4+	
	2	1	CRi/NE/Pos	8.1	LoR	Ena+V+D	CR/NE/Neg	Yes	4.8	SCT
	2	0	CRi/Neg/Pos	19.0	Rising MRD[h]	Ena+V+D	CRi/MRDi/Pos	Yes	11.0+	
	2	0	CR/Neg/Pos	17.0	Rising MRD[i]	Ena+V+D	NR	No	7.0	NR
	1	0	CRi/NE/Pos	3.4	LoR	Ivo+V+A	MLFS	Yes	3.2	LOR
	2	0	CRi/Neg/NE	10.0	Rising MRD	Ena+V+D	CRi/Pos/Pos	Yes	7.1+	
	2	0	CR/Neg/Pos	0.9	MD choice[j]	Ena+V+D	CR/Neg/Neg[k]	Maintained	6.0	Side effects[l]

A azacitidine, *D* decitabine, *DLI* donor lymphocyte infusion, *Ena* enasidenib, *IDHi* IDH inhibitor, *Ivo* ivosidenib, *LoR* loss of response, *LoT* line of therapy, *NE* not evaluable, *Neg* negative, *NR* no response, *Pos* positive, *SCT* stem cell transplant, *SD* stable disease, *ToT* time on therapy, *V* venetoclax.

a. MRD status only reported for CR/CRi responses.

b. Considered a salvage response in the event of a CR/CRi/MLFS/PR.

c. "+" denotes ongoing therapy at time of data cutoff.

d. Novel IDHi received on clinical trial.

e. VEN + HMA discontinued after 1 cycle due to sepsis with multi-organ failure from which the patient recovered and proceeded to enasidenib monotherapy.

f. CRi obtained with enasidenib monotherapy for post SCT relapse, which was converted to a CR following 2 DLIs.

g. Patient discontinued therapy for personal rather than medical reasons with relapse ~2 years post discontinuation. Subsequent CRi obtained upon retreatment with enasidenib monotherapy.

h. Immunophenotype of the rising MRD by flow cytometry was different from that of the original leukemia with a parallel rise in the IDH^{mut} VAF accompanied by progressive cytopenias. Addition of enasidenib reversed this trend (IDH^{mut} VAF 32% > 0.1%, peripheral counts improved).

i. Enasidenib added due to FC-MRD conversion from - to + with accompanying cytogenetic evolution.

j. Once institutional protocol was amended to allow the addition of targeted small-molecule inhibitors.

k. Upon addition of enasidenib to treatment, response deepened to become MRD negative by ddPCR for IDH^{mut}.

l. Gynecomastia which the patient attributed to enasidenib; switched back to VEN + HMA.

Supplemental Table 5. Outcomes with IDH inhibitor-based regimens received directly prior to VEN + HMA (n=8)

	*IDH*mut	No. prior LoT	IDHi regimen	Response IDHi, Marrow/FC-/NGS-MRD*	ToT IDHi regimen, months	Reason for switch to VEN+HMA	Response to VEN+HMA regimen, Marrow/ FC-MR/NGS-MRD*	Did the regimen salvage response post IDHi	ToT VEN+ HMA, months	Reason for VEN+HMA discontinuation
IDHi alone prior	1	1	Ivo	CRi/Pos/NE	9.6	LoR	MLFS	Yes	2.9	SCT
	1†	1	Ivo	CRi/Neg/Pos	10.3	LoR	CRi/Neg/Pos	Yes	19.9	LOR
	2	5	Ena	SD	6.8	NR	CR/Neg/Neg	Yes	2.9	SCT
	2	1	Ena	CR/Pos/Neg	5.2	LoR	MLFS	Yes	3.4	LOR
IDHi doublet prior	2	1	Ena+A	NR	2.1	NR	CR/Neg/Neg	Yes	3.7	SCT
	2	2	Ena+D	NR	1.7	NR	CR/MRDi/Pos	Yes	4.8	SCT
	2	0	Ena+A	MLFS	4.4	LoR	CRi/Neg/Pos	Yes	2.5	Pt choice‡
	2	0	Ena+ICT	NR	3.5	NR	NR	No§	1.0	NR

A azacitidine, *D* decitabine, *Ena* enasidenib, *ICT* intensive chemotherapy, *IDHi* IDH inhibitor, *Ivo* ivosidenib; *LoR* loss of response, *LoT* lines of therapy, *NE* not evaluable, *Neg* negative, *NR* no response, *Pos* positive, *SCT* stem cell transplant, *SD* stable disease, *ToT* time on therapy, *V* venetoclax.

*MRD status only reported for CR/CRi responses.

†Original *IDH*mut was an *IDH1-R132C*, but an *IDH2-R140Q* emerged at time of LOR to ivosidenib.

‡Patient discontinued therapy for personal rather than medical reasons. Relapsed ~ 2 months post VEN + HMA discontinuation.

§The only patient without a MLFS response or better upon switching to VEN + HMA. This patient had a treated secondary AML (antecedent high-risk MDS) with a complex karyotype, NRAS mutation, and VAF-inferred subclonal *IDH2*mut at time of leukemic transformation.

Supplemental Figures

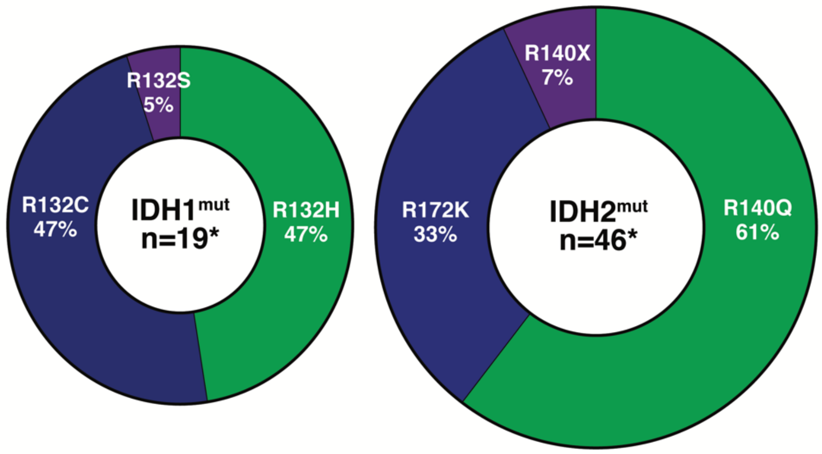

Supplemental Figure 1. *IDH* mutation isotypes prior to VEN + HMA therapy
*The patient with both an *IDH1-R132C* and *IDH2-R140Q* mutation is displayed in the *IDH2*-mutated group given variant allele frequency-inferred clonal dominance.

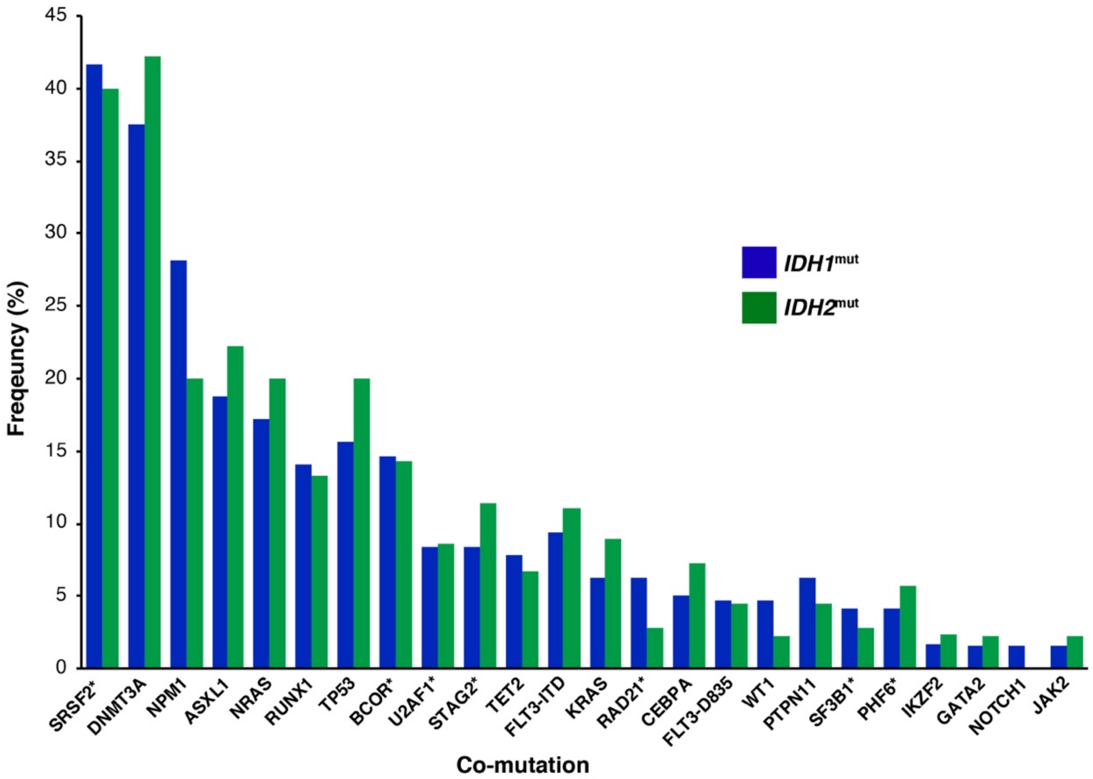

Supplemental Figure 2. Co-mutation frequencies at time of starting VEN + HMA therapy
A pre-treatment comprehensive NGS panel was not evaluable in 1 patient (who underwent isolated *IDH* sequencing) and 16 patient samples were sequenced with less comprehensive earlier-generation NGS panels; therefore, n=64 for genes without an asterix and n=48 for genes denoted by the *.

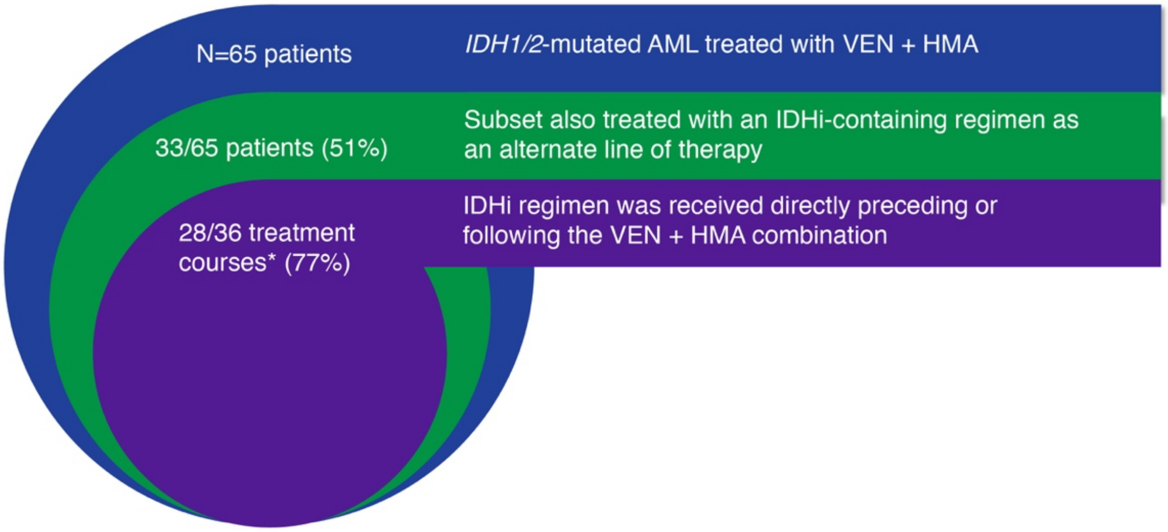

Supplemental Figure 3. Proportion of patients that received an IDHi-containing regimen consecutive to their VEN + HMA treatment course
*3 of the 33 patients who received an IDHi-based regimen each received 2 different regimens at alternate timepoints, accounting for the total of 36 IDHi treatment courses.

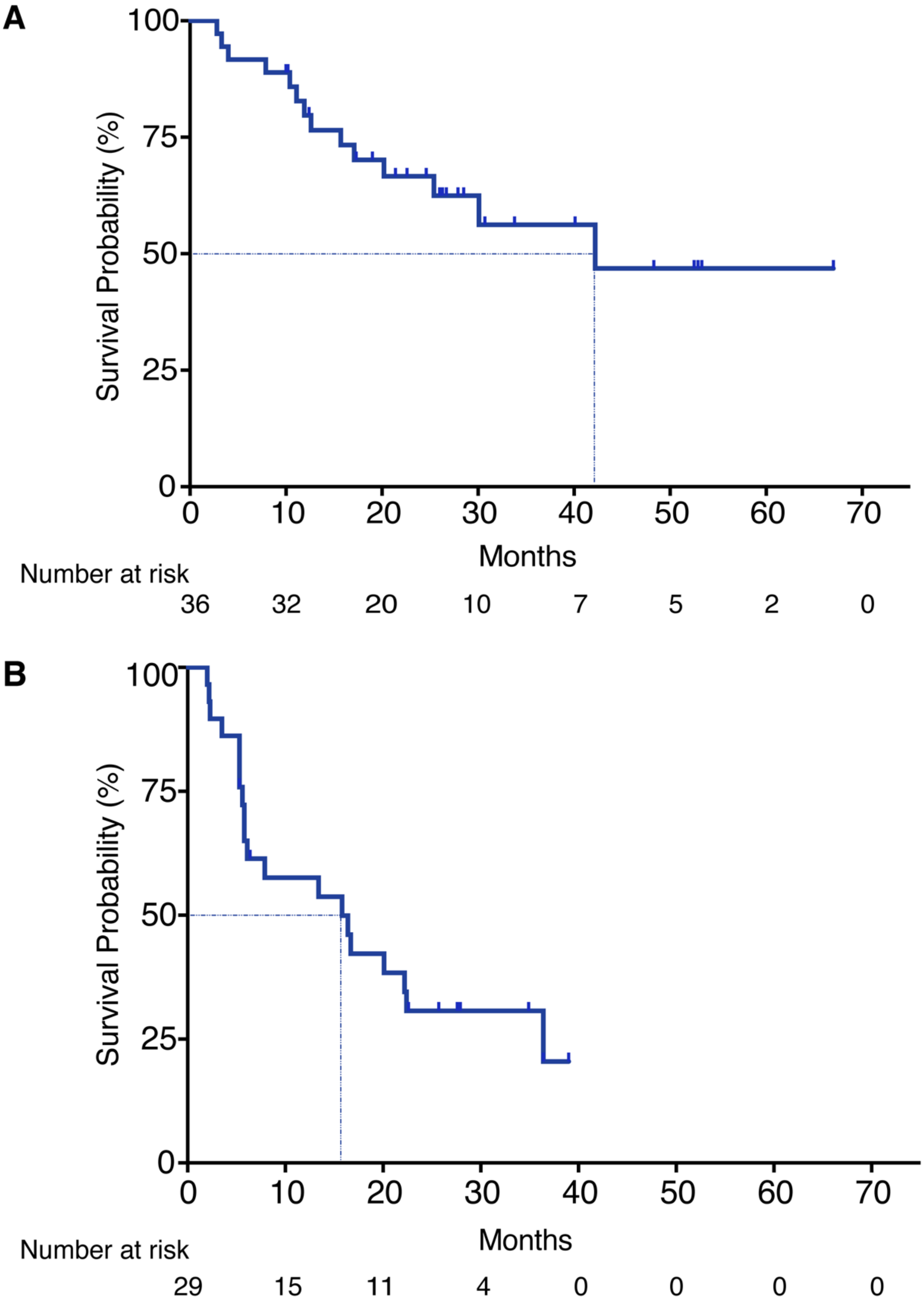

Supplemental Figure 4. Overall survival for patients on VEN + HMA. **A** Frontline and **B** Relapsed and refractory setting. Median OS 42.2 months (95% CI 25.4, NE) and 15.8 months (95% CI 5.8, NE), respectively.

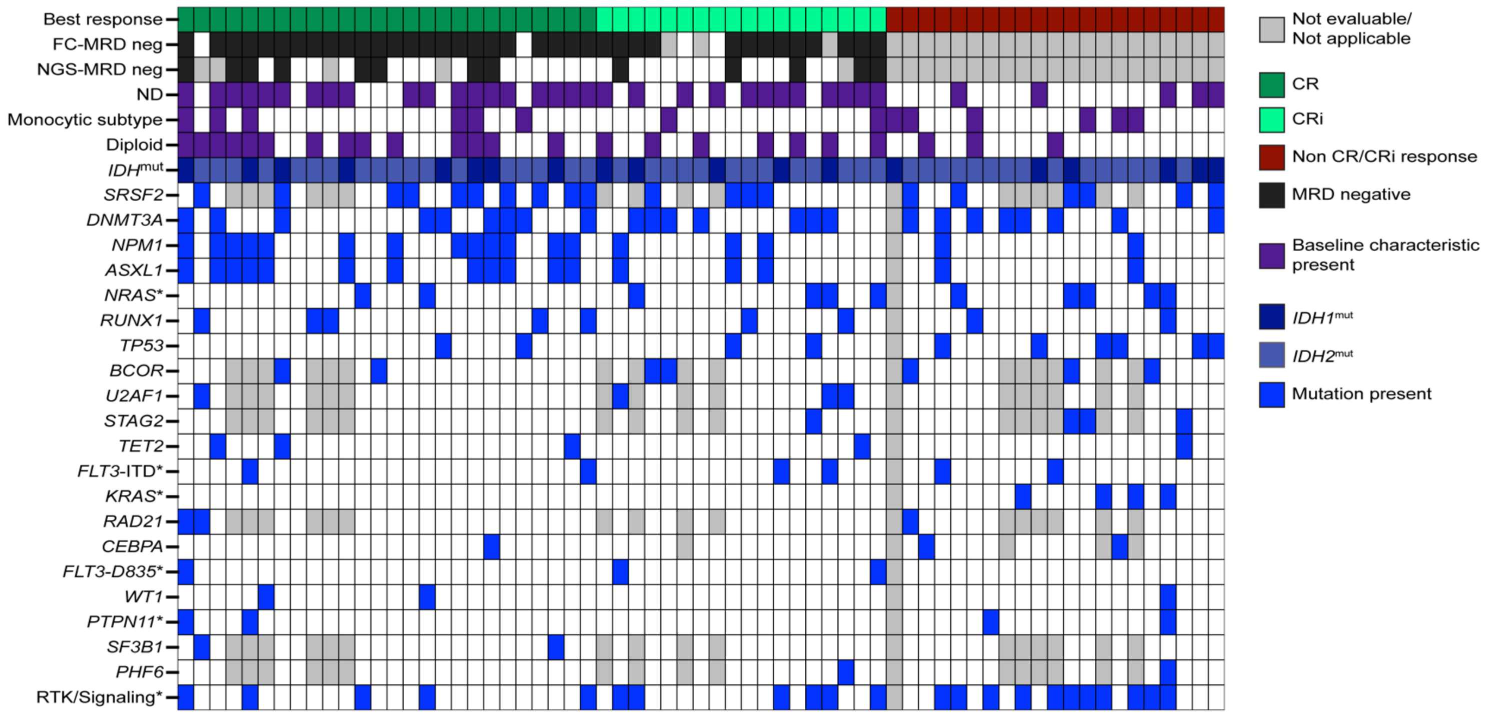

Supplemental Figure 5. Oncoprint displaying response and depth of response by somatic co-mutation landscape. A pre-treatment comprehensive NGS panel was not evaluable in 1 patient (who underwent isolated *IDH* sequencing) and 16 patient samples were sequenced with less comprehensive earlier-generation NGS panels.
CR complete response, *CRi* complete response with incomplete count recovery, *MRD* measurable residual disease, *RTK* receptor tyrosine kinase.
*Genes involved in receptor tyrosine kinase pathways, including members of RAS/MAPK pathway and FLT3 signaling.

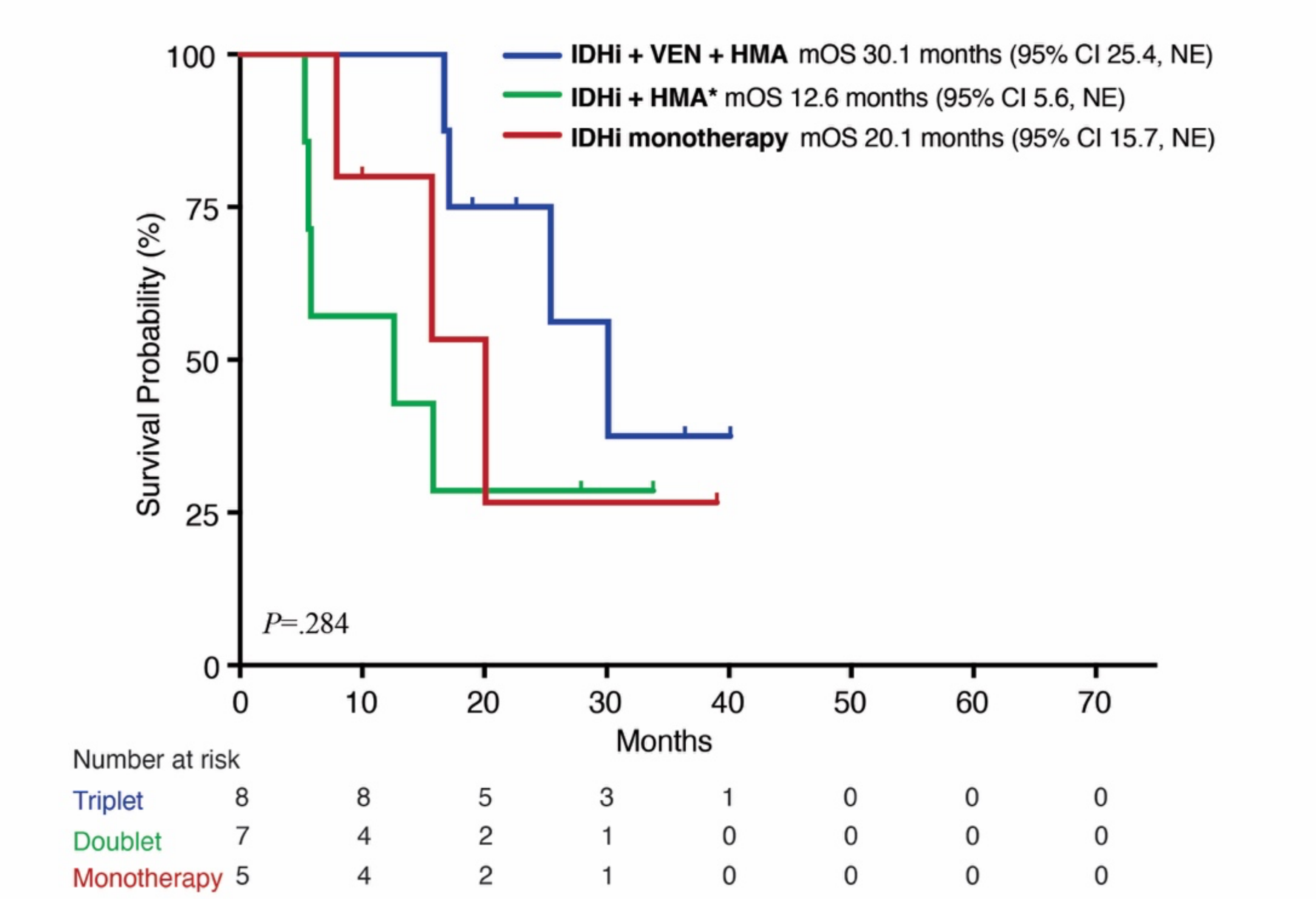

Supplemental Figure 6. Overall survival in the n=20 patients that switched from VEN + HMA to IDHi-based therapies by regimen composition. Survival calculated from C1D1 of VEN + HMA. Includes the 2 patients that transitioned to IDHi-based regimens for reasons other than lack/loss of response.
*One patient in this group received an IDHi in combination with VEN instead of a HMA.
